# On variations of volumetric activity of ^90^Sr and ^137^Cs in the Baltic Sea coastal waters near the shore of Lithuania in 2005–2009

**DOI:** 10.1007/s10967-012-1762-6

**Published:** 2012-04-10

**Authors:** D. Styro, R. Morkūnienė, A. Daunaravičienė

**Affiliations:** Vilnius Gediminas Technical University, Saulėtekio Ave 11, LT-10223 Vilnius, Lithuania

**Keywords:** Artificial radionuclides, Baltic Sea, Coastal waters, Hydro meteorological situations, Volumetric activity

## Abstract

The article presents the measurement results of the volumetric activity (VA) of artificial radionuclides ^90^Sr and ^137^Cs in the coastal waters of the Baltic Sea near the Curonian Peninsula in 2005–2009. The annual average values for this period of time were 12 Bq/m^3^ (^90^Sr) and 40 Bq/m^3^ (^137^Cs). Considerable variations in the VA of the radionuclide in individual measurements compared to the average results were observed. The extreme values were 6 and 16 Bq/m^3^ for ^90^Sr and for ^137^Cs—27 and 75 Bq/m^3^. It is proposed to allow such variations under the influence of a variety of external factors such as hydro meteorological situations, inflowing rivers and bays, storm activity and etc. Besides, a possibility of penetration of radionuclides into the sea waters from the additional radioactive sources is not excluded.

## Introduction

Results of the investigation on artificial radionuclides in the environmental objects provide for the assessment of possible affects of radiation exposure on human health. At the same time investigation of radionuclide distribution in the environment gives valuable information about the on-going processes. Hence it is necessary to consider the basic sources of radioactive pollution, in particular in the Baltic Sea waters, as well as further behaviour of radionuclides under the influence of the external factors.

Artificial radionuclides have penetrated into the Baltic Sea for the first time from the global fallouts as a result of the nuclear and thermonuclear weapon tests in the atmosphere [1]. During a long period of time this sea was contaminated by the North Sea waters where radioactive waste was discharged from various nuclear objects [1].

After the accident in the Chernobyl Power Plant (ChPP) in 1986, an average value of VA of ^137^Cs has increased by an order of magnitude in the Baltic Sea compared to the value which was formed by global fallout (12 Bq/m^3^) [2]. However, an increase of VA of ^137^Cs was observed in the coastal waters during some years after ChPP accident. It is natural that radioactive fallout on the surface waters of the Baltic Sea was inhomogeneous [2].

The biggest amount of ^137^Cs occurred in the northern part of the Baltic Sea and in the Gulf of Bothnia [3]. Therefore, the levelling process of VA of ^137^Cs in the surface waters took place. It means that this radionuclide was transferred from more polluted to less polluted areas, and increased there VA of ^137^Cs.

In particular, the increased values of ^137^Cs VA in the coastal waters of the Baltic Sea in Lithuania were observed up to 1989 and 1990, when its average values were 113 and 117 Bq/m^3^ accordingly [4]. Since 1991 a gradual decrease in average values of VA of ^137^Cs was observed. However, frequent variations in VA values took place because of the secondary effects and also after the changes in the hydro meteorological situation [5]. The variations of VA values of artificial radionuclides after the change of external factors is presented in article [6].

It is known that ^90^Sr practically did not participate in the air mass movement from Chernobyl. Therefore its VA values have changed slightly in the Baltic Sea and a primary source of pollution of this sea by ^90^Sr turned out to be the global fallout. Hence, a gradual decrease of its VA was defined by the radioactive decay. It is determined that VA of ^137^Cs and ^90^Sr in the surface and near bottom waters is almost the same [4, 7].

Since the behaviour of these radionuclides in the sea waters is diverse, their self-purification processes are different also, as well as the variation of VA under the effect of external factors.

Individual observation results of ^90^Sr and ^137^Cs VA demonstrate only an approximate estimation of their behaviour in the sea waters. In order to define their possible variation caused by the natural factors or to find the additional amount of radionuclide which could penetrate into the sea waters from accidental unidentified sources, it is necessary to execute large-scale observations.

There are continuous variations of ^137^Cs and ^90^Sr VA in the Baltic Sea. These facts were registered during the individual experiments and also in the average draft results, which were obtained before and after ChPP accident [8, 9].

In particular since 1991 the VA of the above mentioned radionuclides decreased in the coastal waters of the Baltic Sea near Lithuania. However, obvious variations in the defined VA measurements were observed.

Thus, in 2004 the extreme values of ^137^Cs VA were 94 and 49 Bq/m^3^ [10] and ^90^Sr VA were 17 and 7 Bq/m^3^ [11]. They indicate major changes in the absolute values of the radionuclide VA. According to the average VA of ^137^Cs in the surface waters of the Baltic Sea at a present time and the predicted value [12], almost the same result has place (about 40 Bq/m^3^).

The aim of the present study is to define possible values of variation of ^90^Sr and ^137^Cs VA in coastal waters of the Baltic Sea which can be formed under the influence of natural hydro physical factors.

## Materials and methods

**Fig. 1:**
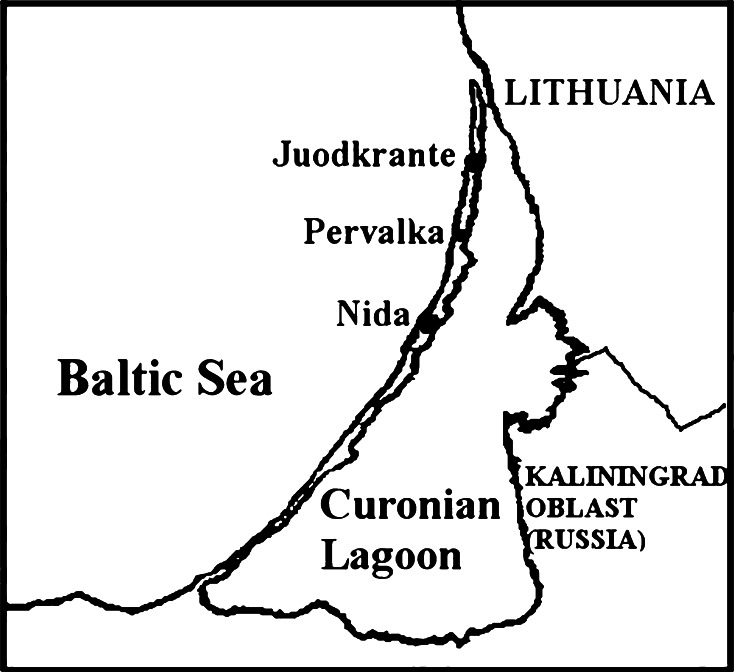
Observation stations

Water samples (40 L) for the determination of ^137^Cs and ^90^Sr volumetric activity (VA) in the near-shore waters of the Baltic Sea (Juodkrante) were taken in 2005–2009 (Fig. 1). In 2008, water samples were taken near Pervalka and Nida. The temperature, specific electric conductivity, water current direction and wind direction were registered during the time of sampling.

**Fig. 2:**
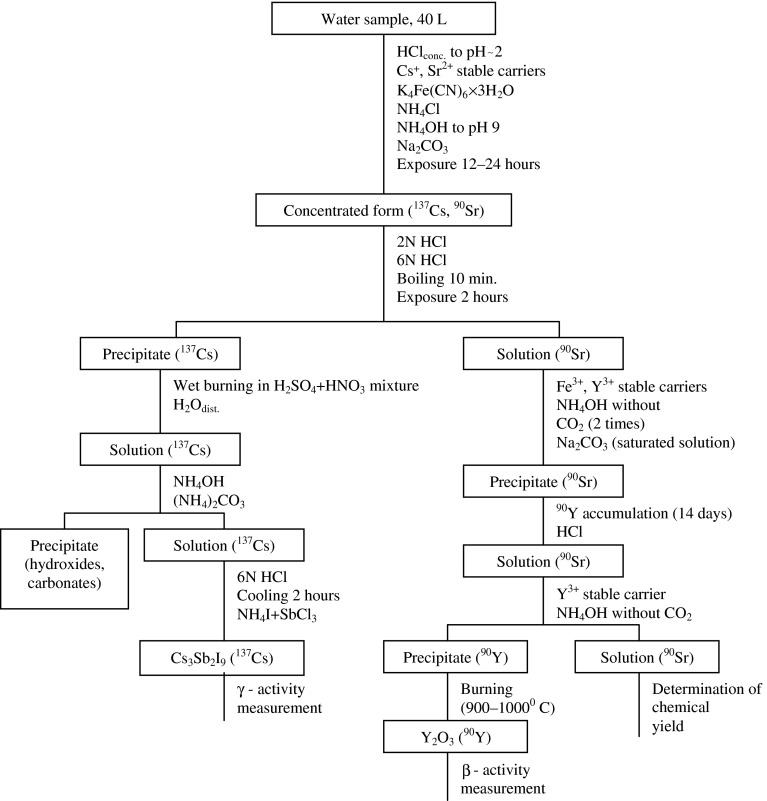
Scheme of radiochemical analysis of ^137^Cs and ^90^Sr

The ^137^Cs and ^90^Sr were concentrated from the same sea water samples after adding the stable carriers by ferrocyanide-carbonate precipitation [11, 13]. The scheme of the radiochemical separation is presented in Fig. 2.

After the radiochemical cleaning the yield of caesium was determined gravimetrically in the form of Cs_3_Sb_2_I_9_. It varied within a range of 60–80 %. The activity of ^137^Cs samples was registered by gamma spectrometer (CANBERRA) with HPGe detector (resolution 2 keV, efficiency 15 %). The determination error for ^137^Cs VA amounted to 10 %. ^90^Sr was measured by ^90^Y emission using a low level background beta radiometer. A stable strontium yield was determined by the atomic absorption spectrometer and Y-gravimetrically in Y_2_O_3_ form. The yield values varied within a range of 60–80 %. The determination error for ^90^Sr VA amounted to 15 %.

## Results

It is known that contamination of the hydrosphere began in 1954, when ^90^Sr was found in the waters of the Atlantic Ocean for the first time [14]. The increase of the global fallout was observed up to 1963, when nuclear and thermonuclear weapon testing countries signed the Treaty Banning Nuclear Weapon Tests in the atmosphere, hydrosphere and outer space. Since then a gradual decrease of radioactive environmental pollution was observed.

However, this process continued up to 1986, i.e. until the ChPP accident. After the accident the radioactive pollution spread far from Chernobyl and in particular toward the Baltic Sea. By that time VA of ^137^Cs has increased in the surface waters by a more than an order of magnitude [3] and nowadays its average VA has decreased 3–4 times. It means that the Baltic Sea is not completely purified from this radionuclide.

During the above period ^90^Sr VA decreased by 15–20 %. This fact can be explained by its negligible transfer to the Baltic Sea after the ChPP accident.

Because of the process of levelling of VA of these radionuclides in the surface water of the Baltic Sea, their absolute values varied in the coastal waters from time to time.

The obtained measurement results near Juodkrante (Fig. 1) in 2005 are presented in Table 1.

**Table 1:** VA of ^137^Cs [15] and ^90^Sr, and data of some hydro meteorological parameters in coastal waters of the Baltic Sea (Juodkrante) in 2005

No.	Date	Conductivity, mS/m	Precipitations, mm	Direction of wind	Velocity of wind, m/s	^90^Sr, Bq/m^3^	^137^Cs, Bq/m^3^
1.	22.04	970	0.0	NW	6	10	62
2.	23.04	965	0.0	N	5	6	69
3.	25.04	990	0.0	E	4	12	74
4.	27.04	990	0.0	E	3	9	66
5.	29.04	1050	0.0	NW	5	13	75
6.	01.05	1050	0.0	S	4	12	75
7.	03.05	1025	2.4	S	6	10	48
8.	05.05	1050	0.0	N	6	14	50
9.	07.05	1025	0.0	W	5	15	56
10.	08.05	1025	0.0	NW	3	16	46
11.	28.06	995	1.7	NW	6	11	45
12.	01.07	900	0.0	W	3	14	54
13.	03.07	900	0.0	–	0	13	55
14.	05.07	900	0.0	E	4	11	54
15.	07.07	960	0.0	E	3	13	54
16.	09.07	960	0.0	E	1	13	54
17.	23.08	990	0.0	E	4	12	49
18.	25.08	930	0.0	S	2	11	50
19.	27.08	960	3.9	SW	6	11	54
20.	29.08	955	0.0	W	5	12	51
21.	31.08	965	0.0	NW	2	10	57
22.	02.09	980	0.0	SW	2	11	48
23.	04.09	980	0.0	E	3	13	47
24.	14.10	880	0.0	W	6	11	46
25.	15.10	920	0.0	N	8	15	41
26.	16.10	940	0.0	N	4	12	46
27.	18.10	960	0.0	NW	3	11	53
28.	20.10	920	0.0	S	6	7	41
29.	22.10	960	1.1	S	6	8	45
30.	23.10	950	11.7	W	7	11	44
31.	25.10	960	8.0	SW	7	15	48
32.	27.10	920	0.0	N	7	13	38
33.	30.10	920	0.0	S	7	10	30
34.	31.10	920	0.0	SW	5	12	27
35.	01.11	920	0.0	SE	6	11	30

According to the obtained results (Table 1), the marked variations of VA of radionuclides have been observed from April to November, 2005.

For ^90^Sr the extreme values for this period of time were 6 and 16 Bq/m^3^ and for ^137^Cs—27 and 75 Bq/m^3^ accordingly; the ratio of the above values was almost the same, i.e. about 2.5. Such variations are possible under the influence of the external factors. However, there is a possibility for the additional penetration of radionuclides from the accidental sources.

**Fig. 3:**
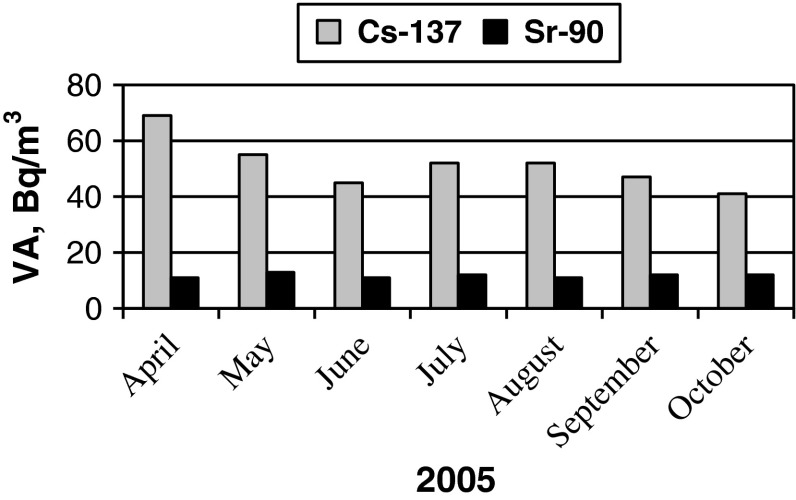
Average values of ^137^Cs and ^90^Sr VA in coastal waters of the Baltic Sea (Juodkrante) in 2005

The average values were 12 Bq/m^3^ (^90^Sr) and 51 Bq/m^3^ (^137^Cs). According to average monthly values, the VA of ^137^Cs has a seasonal course with the highest values in spring and the lowest in autumn (Fig. 3). These results are approximate since various numbers of measures were carried out every month. However, such conformity to natural law concerning VA of ^90^Sr wasn’t observed.

Similar measurements were carried out in April–May, 2006 (Table 2). Then the extreme values of ^90^Sr VA were 9 and 14 Bq/m^3^ and for ^137^Cs—37 and 52 Bq/m^3^. However, the difference between these values is smaller than for the same data in 2005 (Table 1).

**Table 2:** VA of ^137^Cs and ^90^Sr, and data of some hydro meteorological parameters in the coastal waters of the Baltic Sea (Juodkrante) in 2006

No.	Date	Conductivity, mS/m	Precipitations, mm	Direction of wind	Velocity of wind, m/s	^90^Sr, Bq/m^3^	^137^Cs, Bq/m^3^
1.	19.04	980	0.6	W	6	12	37
2.	20.04	980	3.8	S	5	12	37
3.	21.04	990	0.0	NW	2	13	37
4.	22.04	990	0.0	N	6	14	38
5.	25.04	1020	4.2	NW	3	11	39
6.	26.04	990	0.0	NW	4	12	41
7.	27.04	1080	0.0	SE	7	9	42
8.	28.04	1110	0.0	N	2	11	43
9.	29.04	1100	0.0	E	7	10	44
10.	02.05	1100	0.8	S	2	11	44
11.	03.05	1100	0.0	SW	2	13	45
12.	04.05	1080	0.0	N	5	13	45
13.	05.05	1100	0.0	N	4	14	45
14.	06.05	1050	0.0	E	6	11	52

A small number of measurements of VA of ^90^Sr and ^137^Cs were carried out in the Baltic Sea near Juodkrante in 2007. The obtained results are presented in Table 3. Here there is small difference in VA for ^90^Sr and ^137^Cs (since observations were carried out during a short span of time, i.e. from 21st to 27th August, 2007. It is necessary to note, that an average VA of ^90^Sr (12 Bq/m^3^) coincides with the results obtained in 2005 and 2006 (Tables 1 and 2).

**Table 3:** VA of ^137^Cs and ^90^Sr and data of some hydro meteorological parameters in the coastal waters of the Baltic Sea (Juodkrante) in 2007

No.	Date	Conductivity, mS/m	Precipitations, mm	^90^Sr, Bq/m^3^	^137^Cs, Bq/m^3^
1.	21.08	830	1.4	10	45
2.	23.08	920	11.2	14	47
3.	25.08	900	0.0	12	40
4.	27.08	920	4.5	12	47

**Fig. 4:**
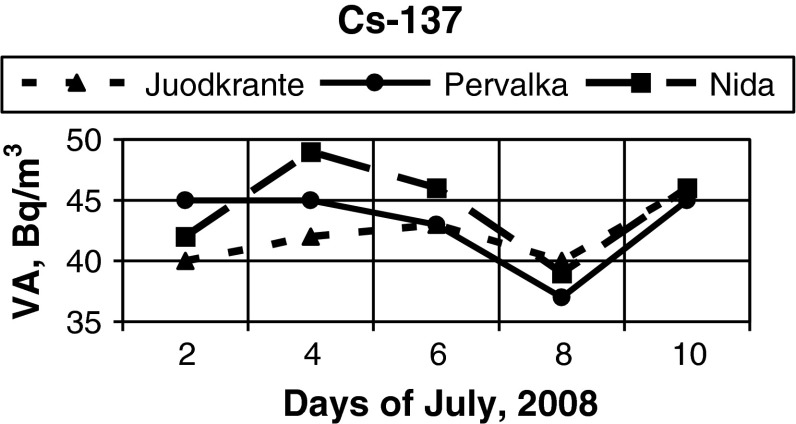
VA of ^137^Cs (Bq/m^3^) in the coastal waters of the Baltic Sea in July, 2008 in observation stations near Juodkrante, Pervalka, Nida (Fig. 1)

**Fig. 5:**
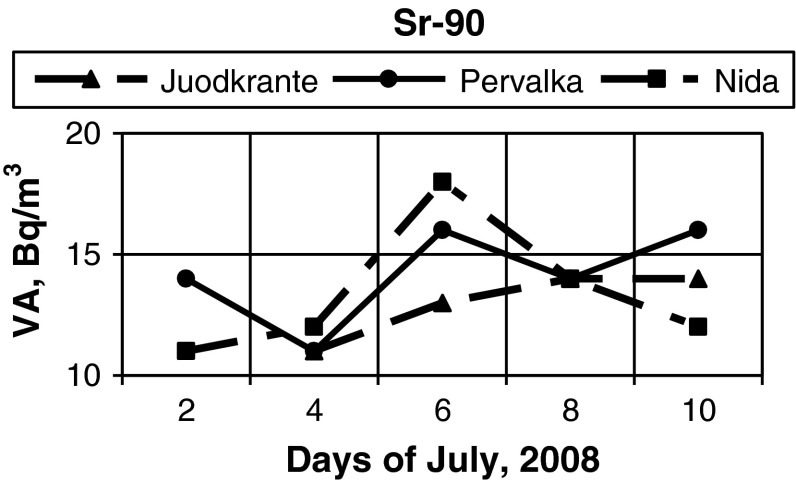
VA of ^90^Sr (Bq/m^3^) in the coastal waters of the Baltic Sea in July, 2008 in observation stations near Juodkrante, Pervalka, Nida (Fig. 1)

Simultaneous observations in VA of radionuclides near Juodkrante, Pervalka and Nida (Fig. 1) were carried out in July, 2008. In some cases a marked difference in VA of radionuclides was observed. The major variations of ^137^Cs VA took place on 4th July (Fig. 4) and ^90^Sr—on 6th July (Fig. 5).

The best coincidence of the obtained results near the above observation stations for ^137^Cs was on 10th July and for ^90^Sr the same results were on 4th and 8th July. These results show that the scale of VA variations in these radionuclides does not coincide in time and can be diverse under identical changes of the external factors. During water sampling minor changes of water conductivity were identified, including the observation of insignificant variations in wind direction and velocity.

The obtained results of radionuclides VA during the second half of August 2009 are presented in Table 4.

**Table 4:** VA of ^137^Cs and ^90^Sr and data of some hydro meteorological parameters in the coastal waters of the Baltic Sea (Juodkrante) in 2009

No.	Date	Precipitations, mm	Direction of wind	Velocity of wind, m/s	^90^Sr, Bq/m^3^	^137^Cs, Bq/m^3^
1.	18.08	9.6	W	9	13	40
2.	19.08	0.0	NW	9	13	43
3.	20.08	0.0	E	2	13	38
4.	21.08	0.0	SW	4	12	37
5.	22.08	1.1	SW	4	14	41

^90^Sr VA practically did not change (Table 4) and its variations did not exceed an experimental error despite the high velocity and changing wind direction. Variation of ^137^Cs VA exceeded an experimental error. Hence, the change of hydro meteorological situations determined different variations of VA of ^90^Sr and ^137^Cs.

**Fig. 6:**
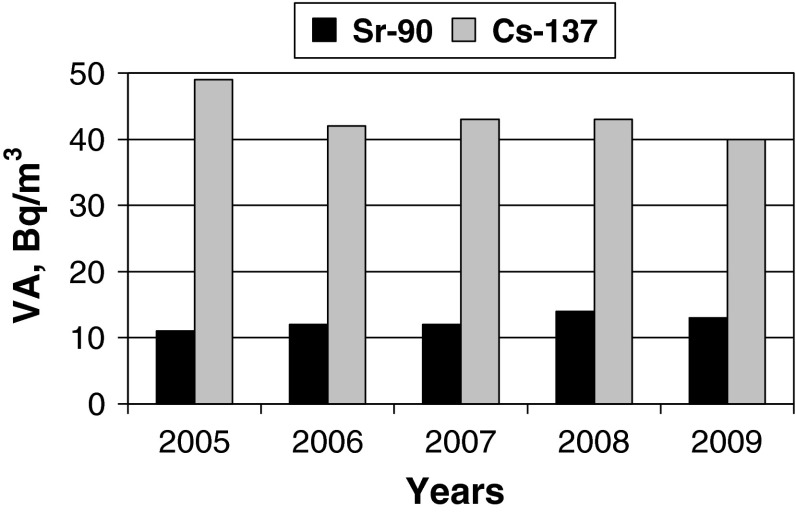
Average values VA (Bq/m^3^) of ^90^Sr and ^137^Cs, obtained in 2005–2009 in the coastal waters of the Baltic Sea (Juodkrante)

The average values of VA of these radionuclides in 2005–2009 are illustrated in Fig. 6. Here a minor increase in the average data of ^90^Sr VA from 11 to 13 Bq/m^3^ was observed from 2005 to 2009. At the same time the average values of ^137^Cs VA decreased from 49 to 40 Bq/m^3^. However, this comparison should be considered as an approximate, since the number of measurements was different from year to year. It is also necessary to focus on the extreme values of the obtained results defining the limits of their possible deviations from average values. This was to be expected that the greatest difference of extreme values was appeared in 2005 when more measurements were carried out. Here the difference in the extreme values of ^90^Sr VA was 10 Bq/m^3^ and the same for ^137^Cs—48 Bq/m^3^. It means that external factors can change VA of the radionuclides to a marked degree.

## Discussion

In the surface waters of the Baltic Sea an average VA of ^90^Sr was 24 Bq/m^3^ and ^137^Cs was 12 Bq/m^3^ [2] before the ChPP accident. After this accident VA of ^90^Sr increased slightly and VA of ^137^Cs increased by more than an order of magnitude. Hence, the process of self-purification of the Baltic Sea waters from these radionuclides has various features. According to theoretical results [12] an average VA of ^137^Cs in the Baltic Sea has to be about 40 Bq/m^3^. Nowadays, however, VA of ^90^Sr was almost the same during the last decade, i.e. 12 Bq/m^3^. ^90^Sr VA decreases because of radioactive decay, but also can increase due to global fallout and other factors.

During 2005–2009, major variations of radionuclides VA were observed and sometimes their absolute values differed from each other more than two times.

However, in order to define the additional amount of radionuclides from other sources of radioactive pollution, it is necessary to know possible variations of VA of radionuclides caused by the natural hydro meteorological situation.

Variations of VA of artificial radionuclides in the coastal waters of the Baltic Sea are possible from inflowing bays and rivers. This causes water mass movement along the coast which depends on wind velocity and direction. Besides, short-term change of radionuclides VA is possible in spring time as a result of the melting snow and surface runoff, i.e. the water flowing over the land surface. During a storm the change in VA of radionuclides is possible as well. It is necessary to take into consideration the intensity and duration of precipitation which can affect the content of radionuclides in the surface sea waters.

After 3rd May, 2005, an obvious decrease of VA of both radionuclides after precipitation (Table 1) was observed. The decrease of radionuclides VA in coastal waters was registered during the last days of October, 2005 when precipitation lasted long enough (Table 1). However, such dependence is not always observed since during precipitation water mass transfer with higher radionuclides VA is also possible.

A short-term increase of radionuclides VA can take place on the sea surface because of the air mass transfer after the forest fires and burning peat lands. It is known, that plants accumulated greater amount of artificial radionuclides after the ChPP accident and global fallouts.

Therefore VA of ^90^Sr and ^137^Cs in the surface waters of the Baltic Sea depends on the season of the year since sea water stratification differs in summer and winter time, and also on the transition period when change of water mass takes place.

Radionuclides from water are especially poorly accepted by the ice cover, whereas in summer time zooplankton, phytoplankton and sea vegetation accumulate radionuclides. Thus, a change in artificial radionuclides VA is possible because of the above processes. It is necessary to note that external effects on the Baltic Sea waters not always cause the identical tendency of ^90^Sr and ^137^Cs VA change since these radionuclides behave differently in the sea waters. However, extreme variations of radionuclides VA caused by the natural factors have their limits.

## Conclusions

Average values of ^90^Sr and ^137^Cs VA decrease in time because of their natural radioactive decay and the decrease of the environmental radioactive pollution. Nowadays an average VA of ^90^Sr has to be considered 12 Bq/m^3^ and ^137^Cs—40 Bq/m^3^ in coastal waters of the Baltic Sea near Lithuania. Compared to these values the variations of radionuclides VA exceeded them by 50 % in 2005 when long-term observations took place.

Marked variations of radionuclides VA during short-term observations in 2006–2009 were not identified. Therefore, the additional measurement results are necessary to define the limited values of VA of ^90^Sr and ^137^Cs. More measurements must be executed in order to determine the effect of new radioactive sources.

Hence, the present results have to be considered as preliminary.

## References

[1] Styro D (1989). Nuclear hydro physics problems.

[2] Styro D, Bumeliene Zh, Kadzhiene G (1990). Artificial radionuclides volumetric activity fields structure in the surface waters of the Baltic Sea in autumn 1986–1987. Atomnaya Energiya.

[3] HELCOM (2003) Baltic Sea environment proceedings. No. 85. Helsinki Commission, Helsinki

[4] Styro D, Bumeliene Zh, Lukinskiene M, Morkuniene R (2001). ^137^Cs and ^90^Sr behavioural regularities in the south-eastern part of the Baltic Sea. J Environ Radioact.

[5] Styro D, Lukinskienė M, Morkūnienė R (2002). Comparison of water self-cleaning from the radionuclides ^137^Cs and ^90^Sr in the southeastern area of the Baltic Sea. Environ Eng.

[6] Zalewska T, Lipska J (2006). Contamination of the eastern Baltic Sea with ^137^Cs and ^90^Sr over the period 2000-2004. J Environ Radioact.

[7] HELCOM (2009) Baltic Sea environment proceedings. No. 117. Helsinki Commission, Helsinki

[8] Ikäheimonen TK, Outola I, Vartti VP, Kolilainen P (2009). Radioactivity in the Baltic Sea: inventories and temporal trends of ^137^Cs and ^90^Sr in water and sediments. J Radional Nucl Chem.

[9] Lujanienė G, Baneš P, Štamberg K (2010). Experimental study and modeling of ^137^Cs sorption behaviour in the Baltic Sea and the Curonian Lagoon. J Radional Nucl Chem.

[10] Styra D, Kleiza J, Morkūnienė R, Daunaravičienė A (2006). Change cyclicity of volumetric activity of radionuclide ^137^Cs in coastal waters of the Baltic Sea and its possible reasons. J Environ Eng Landsc Manag.

[11] Styro D, Morkuniene R, Daunaraviciene A (2011) Radionuclide ^90^Sr volume activity variations at the Baltic Sea coast near Juodkrante. In: Selected papers of the 8th international conference “environmental engineering”, vol 1. Vilnius Gediminas Technical University Press “Technika”, Lithuania, pp 357–361

[12] Styra D, Bumelienė Ž, Kleiza J (2000). Prognosis of the Baltic Sea self–cleaning from the “Chernobyl” radionuclide ^137^Cs. Aplinkos inžinerija.

[13] Styra D, Morkuniene R, Daunaraviciene A (2008) Self-purification process of the Baltic Sea from ^137^Cs radionuclide in 1986-2006. In: Selected papers of the 7th international conference “environmental engineering”, vol 1. Vilnius Gediminas Technical University Press “Technika”, Lithuania, pp 362–367

[14] Nelepo BA (1970). Nuclear hydro physics.

[15] Styro D, Morkūnienė R, Astrauskienė N, Daunaravičienė A (2007). Comparison and analysis of investigation results on volumetric activity of ^137^Cs at the Baltic Sea coast in 2003–2005. J Environ Eng Landsc Manag.

